# Clinicopathological Profile and Outcome of Childhood Urticaria Vasculitis: An Observational Study

**DOI:** 10.7759/cureus.11489

**Published:** 2020-11-15

**Authors:** Amit Satapathy, Basudev Biswal, Lipsa Priyadarshini, Chandrasekhar Sirka, Lipsa Das, Pritinanda Mishra, Sujata Patodia, Subhashree Kar, Samarendra Mahapatro, Rashmi R Das

**Affiliations:** 1 Pediatrics, All India Institute of Medical Sciences, Bhubaneswar, IND; 2 Dermatology, All India Institute of Medical Sciences, Bhubaneswar, IND; 3 Pathology, All India Institute of Medical Sciences, Bhubaneswar, IND

**Keywords:** urticaria, vasculitis, hypersensitive reaction, steroid, anti-histamine, pediatrics

## Abstract

Background

Urticaria is a type III hypersensitivity reaction usually triggered by an infection, medication, or food item. It usually subsides within 24 hours without any residual lesion and does not have any systemic manifestation. Urticaria vasculitis (UV) is a clinicopathological condition defined by the presence of an urticarial lesion lasting for >24 hours or recurrent episodes of urticaria associated with histopathological features of leukocytoclastic vasculitis.

Methods

This retrospective study was conducted in a tertiary care teaching institute in Eastern India over a period of 2 and ½ years. Children presenting with urticaria lesions for a duration of > 24 hours that did not subside either spontaneously or with anti-histamines were admitted for further workup and management.

Results

During the study period (July 2015 to December 2017), a total of 20 children with urticaria needed admission for symptom control and further workup. There were 16 boys and 4 girls. The mean (SD) age of presentation was 6.5 years (±2.4). Besides urticaria in all, pain abdomen was present in 13 (65%) and fever in 6 (30%) children. Only one had arthritis. Skin biopsy performed in these children was suggestive of leukocytoclastic vasculitis. One child was ANA (anti-nuclear antibody) positive with low C3. All except three children required systemic steroid for symptom control along with other medications (anti-histamines). None had suffered any complication or relapse.

Conclusions

Urticaria vasculitis (most common cause being idiopathic) remains underdiagnosed because of the need of confirmation by biopsy, which might not always be attempted in every case. Though anti-histamines remain the main stay of treatment, adding short course oral steroid shortens the course once infection is ruled out.

## Introduction

Urticaria is a self-limiting cutaneous condition presenting with transient, erythematous, and pruritic wheals with or without angioedema [1-4]. It is a type III hypersensitive reaction usually triggered by infection and by hypersensitivity to medications or food items. Individual lesion in acute urticaria subsides within 24 hours of appearance without any residual lesion and does not have any systemic manifestation. Urticaria vasculitis (UV) is a clinicopathological condition defined by the presence of an urticarial lesion lasting for >24 hours or recurrent episodes of urticaria associated with histopathological features of leukocytoclastic vasculitis [1-4]. Like UV, serum sickness-like syndrome (SSLS) is another condition where lesions last for more than 24 hours. UV predominantly affects the post-capillary venule [1-4]. Though common in adults, it is rare in children. In this retrospective study, we describe the clinical profile and outcome of children presenting with urticaria and UV.

## Materials and methods

This retrospective study was conducted in a tertiary care teaching hospital in Eastern India over a period of 2 and ½ years (July 2015 to December 2017). Children presenting with urticarial lesions for more than 24 hours that did not get subside either spontaneously or with anti-histamines were admitted for further workup and management. Detailed history was taken, and thorough physical examination was performed. The details of demographic factors, clinical presentations (rash and other systemic symptoms such as fever, pain abdomen, joint pain, urinary and eye problems), laboratory data (complete blood count, urine routine and microscopy, ESR [erythrocyte sedimentation rate], C-reactive protein [CRP], anti-nuclear antibody, rheumatoid factor, anti-double stranded DNA antibody, and viral markers such as HIV, HBsAg [hepatitis B surface antigen], anti-HCV [hepatitis C virus]), and other relevant information were recorded in a predesigned pro forma. Skin biopsy was performed for confirmation of the diagnosis by a trained dermatologist. Symptomatic treatment was given or continued with anti-histamines (hydroxyzine and levocetirizine suspension or tablet), antipyretics (paracetamol suspension or tablet), or anti-spasmodics (dicyclomine hydrochloride/drotaverine suspension or tablet). When symptom was severe (pain abdomen, burning or painful, severe itching, and/or presence of systemic symptoms) or not controlled with any of these agents, a short course of systemic steroid (oral prednisolone suspension or tablet) was given at a dose of 1 mg/kg/day (maximum 30-40 mg/day) for seven days. Follow-up was done over a period of six months to one year. Institutional Ethics Committee Approval was taken. Data are presented as descriptive statistics.

## Results

During the study period, 20 children with urticaria were admitted for symptom control and further workup. There were 16 boys and 4 girls. The mean (SD) age of presentation was 6.5 years (±2.4). The detailed characteristics of the included children have been described in Table 1.

**Table 1:** Demographic, clinical, and laboratory data of the study population (n = 20) *Data are presented as number (%) unless specified otherwise. ANA, anti-nuclear antibody; C3, complement component 3; CRP, C-reactive protein; ESR, erythrocyte sedimentation rate; Hb, hemoglobin; RTI, respiratory tract infection; SD, standard deviation; TLC, total leukocyte count; UTI, urinary tract infection

Demographic, clinical, and laboratory data	Number (%)*
Age (year), mean (SD)	6.5 (2.4)
Boys	16 (80)
Duration of symptom (days), median (range)	3 (2–5)
Skin lesions	20 (100)
Fever	6 (30)
Pain abdomen	13 (65)
Joint pain	1 (5)
Complete blood count, mean (SD)	
Hb (g/dL)	11.8 (1.5)
TLC (per cumm)	14,373 (6,054)
Platelet count (10^5^ per cumm)	4.06 (1.5)
ESR (mm/hour), mean (SD)	33 (16)
CRP (qualitative) (>6 mg/dL)	20 (100)
ANA +ve	1 (5)
C3 low	1 (5)
Normal urine microscopy (no hematuria/proteinuria)	20 (100)
Preceding infection	
RTI	1 (5)
UTI	2 (10)

Clinical features

The clinical features have been detailed in Tables 1, 2. The included children had typical popular, erythematous urticaria lasting for more than 24 hours at presentation, with an average duration of 72 hours. After observation, in seven cases the individual lesions persisted for >24 hours, and in rest, the lesions recurred or continue to evolve beyond 24 hours. The typical lesions were plaque-like erythematous lesions present all over the body, including faces, and were pruritic. Pain in the abdomen was seen in 13 (65%) children and fever was seen in 6 (30%) children. None of the children had urinary complaints or blood in stool. Ultrasound of the abdomen performed in selected cases was normal. Only one child had features of arthritis.

**Table 2:** Clinical presentation, C3/ANA level, treatment, and outcome (n = 20) ANA, anti-nuclear antibody; C3, complement component 3; D, day; DOP, day of presentation; DOR, day of resolution of urticaria; F, female; M, male

Age (year)	Sex	DOP	DOR	Systemic symptom	C3/ANA	Oral steroid	Relapse
7.6	M	D1	D4	Pain in the abdomen, fever	-	-	-
7	M	D2	D3	Pain in the abdomen	-	Yes	-
6	M	D2	D4	Pain in the abdomen, fever	-	Yes	-
2.5	M	D2	D4	Fever	-	-	-
5.5	F	D1	D3	Pain in the abdomen	-	-	-
4	F	D1	D3	Pain in the abdomen	-	Yes	-
9	M	D1	D4	Fever	-	Yes	-
7	M	D1	D3	Pain in the abdomen	-	Yes	-
14	M	D5	D8	Pain in the abdomen, arthritis	ANA++ C3 low	Yes	-
4	M	D3	D6	Pain in the abdomen	-	Yes	-
7	M	D2	D3	Pain in the abdomen, fever	-	Yes	-
6	M	D3	D6	Pain in the abdomen	-	Yes	-
9	M	D5	D8	Pain in the abdomen	-	Yes	-
7	M	D4	D6	Pain in the abdomen	-	Yes	-
5.5	M	D3	D6	-	-	Yes	-
3	M	D4	D7	-	-	Yes	-
7	M	D3	D7	Pain in the abdomen	-	Yes	-
5.3	F	D2	D5	Fever	-	Yes	-
6.6	M	D4	D6	Pain in the abdomen	-	Yes	-
8	F	D3	D5	Fever	-	Yes	-

Laboratory investigations

Laboratory investigations have been detailed in Tables 1, 2. All the children (except one) were noted to have raised ESR (mean: 33 mm/hour; SD: 16) and CRP. The mean (SD) hemoglobin was 11.8 g/dL (1.5), total leukocyte count was 14,373 per cumm (6,736), and platelet count was 4.06 x 10^5^ per cumm (1.5). None had a documented hematuria or proteinuria. Only one child had ANA positive, whereas rest all had ANA negative with normal C3 and rheumatoid factor. Skin biopsy of the cases done was suggestive of leukocytoclastic vasculitis.

Treatment and outcomes

Treatment and outcomes have been detailed in Table 2. All children had received anti-histamines over a period of 24-48 hours before presenting to us. Nearly 50% children required systemic steroids for symptom control. The usual time of response to treatment was 24-48 hours after initiation of steroid. None had any deterioration after starting steroid (oral prednisolone: 1-1.5 mg/kg/day, five to seven days). Oral antibiotics were prescribed in 15% children for respiratory and urinary tract infection (UTI); in these children, lesions disappeared without any recurrence. The skin lesions healed in all children without any residual hyperpigmentation. There was no relapse on follow-up at one year.

## Discussion

Urticaria is an annular cutaneous erythematous lesion presenting as a wheal and/or angioedema. In acute urticaria, the lesion usually subsides within 24 hours of appearance (with or without the requirement of an anti-histaminic); in recurrent acute urticaria, the lesions may recur over a period of less than six weeks [2]. These are usually not associated with any systemic manifestations in the form of severe pain abdomen or fever or vomiting. The most common etiologies for acute urticaria are food, drugs, infection (both viral and bacterial), insect bite, and chemicals [3,4]. However, in this study, there was no history of any food or drug intake that could have triggered the urticaria. Irrespective of etiologies, the pathogenesis of urticaria includes mast cell degranulation and release of preformed mediators, such as histamine, and prostaglandin, leading to vasodilatation and increase in endothelial permeability. [5]. The typical histopathological features are dermal edema with perivascular infiltrates with lymphocytes and eosinophils, and some neutrophils [6]. Anti-histamines are the main stay of treatment. First-generation anti-histamines (hydroxyzine and diphenhydramine) are the agent of choice, though second-generation anti-histamines (cetirizine, loratadine, and fexofenadine) can also be used as alternate drugs with lesser adverse effects [7].

In acute urticaria with individual lesion lasting for more than 24 hours, alternative diagnosis must be considered. The common differential diagnosis for these lesions is UV. It is a variant of leukocytoclastic vasculitis involving post-capillary venules [8]. The incidence of UV ranges from 2% to 40% in adults, whereas there is a dearth of data on its incidence in children [9-12]. It is often underreported because of the strict diagnostic criteria, which may not always be present. It usually presents with urticarial rash lasting > 24 hour (mean duration: three to four days) and leaves behind hyperpigmentation on resolution [12]. It is associated with systemic features such as fever, gastrointestinal features, arthritis, and, rarely, nephritis [11]. It may be normocomplimentemic or hypocomplimentemic, depending on C3 level [13]. Normocomplimentemic UV has better prognosis, whereas hypocomplimentemic has a higher chance of relapse. But in our study, none of the children had relapse. The diagnosis of UV is based on typical histological features on skin biopsy suggestive of perivascular inflammatory infiltrates (neutrophils, and eosinophils), leukocytoclasia, and fibrinoid necrosis [14]. Treatment modalities include anti-histamines, NSAIDs (non-steroidal anti-inflammatory drugs), and systemic steroid. Rarely, azathioprine, mycophenolate mofetil, and other immunosuppressive drugs may be required in severe cases [15].

Another etiology for such lesions is SSLS. It clinically presents as urticarial skin lesion with systemic manifestation in the form of fever, arthritis, and lymphadenopathy [16]. It is due to hypersensitivity reaction to drugs (commonly cefaclor, anticancer drugs, and anticonvulsants) [17]. They usually manifest after 7-21 days of exposure and remain for 3-4 days. Laboratory findings include leukocytosis with elevated ESR and CRP. Skin biopsy revealed perivascular and dermal infiltrates with inflammatory cells (such as neutrophils, lymphocytes) without any fibrinoid necrosis [17,18]. Management includes discontinuation of the offending agent and symptomatic treatment with anti-histamines. In our series, though all children had systemic manifestations in the form of pain abdomen and fever, none had typical histological features suggestive of SSLS.

In the study, predominant systemic manifestations were pain abdomen (80%) and fever (30%), with arthritis seen in only one child. In chronic urticaria, systemic manifestations such as joint pain or swelling (55.3%), headache/fatigue (47.6%), flushing (42.7%), gastrointestinal complaints (26.2%), and palpitations (9.7%) have been described, though these data in the literature are lacking in acute urticaria [19]. But systemic manifestations are common in both acute and chronic UV. Almost 68% of adults with hypocomplimentemic UV had systemic symptoms in a previously published study, and acute-phase reactants (e.g., ESR) were elevated in almost all the cases [20]. All but one had normal C3 and ANA. The lesions did not have any hyperpigmentation on healing, which is typical of UV as described in the literature. 

Infection was documented in only three children (one with respiratory tract infection, and the other two with UTI). We could not document any other etiology in our children. Seventeen of our children had received systemic steroid for short duration, with rapid resolution of symptoms. None of the children had any worsening after starting steroid. Average duration of relieve of symptoms was 48-72 hours. Early relief of the symptoms helped in quick improvement of general condition in the present series.

## Conclusions

UV, with the most common cause being idiopathic, remains underdiagnosed because of the need of confirmation by biopsy, which might not be always attempted in every case. Though anti-histamines remain the main stay of treatment, adding short course oral steroid shortens the course once infection is ruled out. All the children recovered well without relapse.
